# Preoperative serum albumin is associated with intra‐abdominal infection following major hepatectomy

**DOI:** 10.1002/jhbp.673

**Published:** 2019-10-15

**Authors:** Narongsak Rungsakulkij, Watoo Vassanasiri, Pongsatorn Tangtawee, Wikran Suragul, Paramin Muangkaew, Somkit Mingphruedhi, Suraida Aeesoa

**Affiliations:** ^1^ Department of Surgery Faculty of Medicine Ramathibodi Hospital Mahidol University 270 Rama VI Road, Ratchathewi Bangkok 10400 Thailand

**Keywords:** Hepatectomy, Intra‐abdominal infection, Risk factors, Serum albumin, Treatment outcome

## Abstract

**Background:**

Major hepatectomy is a complex surgical procedure with high morbidity. Intra‐abdominal infection (IAI) is common following hepatectomy and affects treatment outcomes. This study was performed to investigate perioperative factors and determine whether the preoperative serum albumin level is associated with IAI following major hepatectomy.

**Methods:**

From January 2008 to December 2018, 268 patients underwent major hepatectomy. We retrospectively analyzed demographic data and preoperative and perioperative variables. IAI was defined as organ/space surgical site infection. Risk factors for IAI were analyzed by logistic regression analysis.

**Results:**

In total, 268 patients were evaluated. IAI was observed in 38 patients (14.6%). The mortality rate in the IAI group was 15.7%. Multivariate logistic analysis confirmed that the serum albumin level (odds ratio 0.91; 95% confidence interval 0.84–0.97; *P* = 0.03) and operative duration (odds ratio 1.50; 95% confidence interval 1.18–1.91; *P* < 0.01) were independent factors associated with IAI. A logistic model using the serum albumin level and operative duration to estimate the probability of IAI was analyzed. The area under the receiver operating characteristic curve for predicting IAI was 0.78.

**Conclusion:**

The serum albumin level and operative duration were independent factors predicting IAI following major hepatectomy.

## Graphical abstract

***Highlight***

Rungsakulkij and colleagues set out to investigate the as yet controversial association between preoperative serum albumin levels and intra‐abdominal infection following major hepatectomy. Preoperative serum albumin levels and operative duration were predictors of postoperative intra‐abdominal infection, indicating the need for preoperative nutritional intervention in patients with low serum albumin levels.

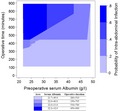

## Introduction

Hepatectomy is the standard treatment for both benign and malignant diseases of the liver with adequate functional reserve 1, 2. The mortality rate of hepatectomy is very low in high‐volume centers 2, 3, 4. Despite technical advances, improvements in perioperative management, a greater understanding of the physiology of the liver, and accumulation of experience with liver resection at specialized centers, the postoperative morbidity rate is still relatively high at up to 40% according to previous reports 3, 5. Major hepatectomy is a complex surgical procedure and has a higher complication rate than minor surgery 5, 6. Although no consensus has been reached regarding the definition of major hepatectomy, the removal of more than three liver segments is the definition generally accepted worldwide 6, 7.

Surgical site infection (SSI) is a common complication following hepatectomy and is classified as superficial SSI, deep or incisional SSI, and organ/space SSI 8. Organ/space SSI and intra‐abdominal infection (IAI) are lethal complications following hepatectomy, and previous studies have shown that the incidence of IAI following hepatectomy may reach 17% 9, 10, 11, 12. IAI is associated with poor long‐term outcomes in patients with both primary and secondary malignancies following hepatectomy 13, 14, 15. Various preoperative and perioperative factors are associated with IAI following hepatectomy. Preoperative risk factors for IAI include the body mass index, serum albumin level, smoking, dialysis, serum bilirubin level, anemia, and repeat hepatectomy. The operative duration, extent of surgery, bile leakage, concomitant bowel surgery, and blood loss are perioperative risk factors for IAI 12. The extent of hepatic resection is strongly associated with the development of IAI. Kenjo et al. 2 and Dokmak et al. 3 reported higher morbidity rates in patients undergoing major hepatectomy than minor hepatectomy.

The preoperative serum albumin level is strongly associated with outcomes following various types of gastrointestinal surgery, including gastric, pancreatic, and colorectal surgery 16, 17, as well as non‐gastrointestinal surgery 18. The association between serum albumin and IAI is controversial 2, 11, 12. To the best of our knowledge, no study has yet been performed to evaluate the association of the preoperative serum albumin level with IAI in patients undergoing major hepatectomy. The present study was performed to investigate perioperative factors and determine whether the preoperative serum albumin level is associated with IAI following major hepatectomy.

## Methods

A total of 268 consecutive patients underwent major hepatectomy at the Department of Surgery, Ramathibodi Hospital, Mahidol University, Bangkok, Thailand from January 2008 to December 2018, and their data were retrospectively analyzed. The study protocol was approved by the Institutional Ethical Committee at Faculty of Medicine, Ramathibodi Hospital, Mahidol University, Thailand (protocol number, MURA2019/80). Major hepatectomy was defined as removal of four or more segments. The liver segments were defined according to the Brisbane classification 19. All patients underwent preoperative cross‐sectional dynamic imaging using either triple‐phase computed tomography or magnetic resonance imaging. Routine blood examinations included a complete blood count, coagulogram, liver and kidney function tests, and the preoperative serum alpha‐fetoprotein level. The low serum albumin was defined as serum albumin level <35 g/l. Preoperative characteristics including the American Society of Anesthesiologists (ASA) class, evidence of hepatitis virus B or C, and smoking were recorded. The preoperative nutritional status was assessed from the Nutritional Alert Form (NAF). The patients were classified to A (normal‐mild malnutrition), B (moderate malnutrition), C (severe malnutrition) 20. Preoperative indocyanine green retention test at 15 min (ICG‐R15) was also performed. Preoperative biliary intervention was defined as the performance of preoperative percutaneous biliary drainage, endoscopic biliary drainage, and previous operative procedures involving the biliary tract. The indication for preoperative biliary intervention is cholangitis and for preoperative biliary drainage in the patients who had perihilar obstruction. Makuuchi’s criteria are used to select patients for curative resection in our center 21. The extent of liver resection was based on the patient’s liver functional reserve as assessed mainly by Makuuchi’s criteria, including the preoperative ascites volume, Child–Pugh score, ICG‐R15 value, and occasionally, volumetric computed tomography analysis. A preoperative evaluation was conducted by a multidisciplinary team including a surgeon, gastroenterologist, medical oncologist, and interventionist every week at our hospital. The platelet‐to‐lymphocyte ratio (PLR), prognostic nutritional index (PNI) and neutrophil‐to‐lymphocyte ratio (NLR) are among the preoperative serum inflammatory indices included in the study. PLR was calculated as the platelet count divided by the lymphocyte count. PNI was calculated as albumin (g/l) + 0.005 × total lymphocyte count (/µl). NLR was calculated as neutrophil count divided by the lymphocyte count.

### Perioperative method

Prophylactic antibiotics were routinely given with intravenous cefazolin or cefoxitin 1.0 g within 30 min before skin incision. The incision type depended on the surgeon’s preference. The Pringle maneuver was performed using intermittent clamping. Intraoperative ultrasound was routinely used to stage the disease and to guide parenchymal transection, which was performed using a Cavitron ultrasonic aspirator, the clamp–crushing technique, or a combined technique depending on the surgeon’s preference. A prophylactic drainage tube was routinely placed. The suture material was also dependent upon the surgeon’s preference. Blood loss was estimated by an anesthesiologist, who also assessed the need for blood transfusion. The operative time was defined as the period from the start of the incision to closure of the abdominal wound.

### Postoperative complications

Intra‐abdominal infection was defined as an infection that occurred within 30 days postoperatively, appeared to be related to the operation and involved any part of the anatomy other than the incision that was opened or manipulated during the operation, and showed at least one of the following characteristics: purulent drainage from the tube placed through a stab wound into the organ space; isolation of organisms from an aseptically obtained culture of fluid or tissue from the organ space; development of an abscess or other evidence of infection involving the organ space as found on direct examination, during reoperation, or by histopathologic or radiologic examination; or diagnosis of an organ/space SSI made by the surgeon or attending doctor 8. Bile leakage was classified as grade A, B, and C according to the International Study Group of Liver Surgery 22. The drainage tube was removed after postoperative day 3. The patients who had bile leakage, the drainage tube was placed until there was no evidence of bile leakage. The IAI patients who had sepsis, the reoperation was performed. Postoperative mortality was recorded as 90‐day mortality and in‐hospital mortality.

### Statistical analyses

Among the patient characteristics, continuous variables were compared by Student’s *t*‐test and categorical variables were compared by the χ^2^ test or Fisher’s exact test. A *P*‐value of <0.05 was considered statistically significant. The potential risk factors were analyzed by univariate and multivariate methods using a logistic regression model. Independent risk factors were expressed as odds ratio (OR) with 95% confidence interval (CI). The performance of the predictor for the variable factor was analyzed by the receiver operating characteristic (ROC) curves.

Internal validity of the final model’s performance in terms of area under the curve (AUC) of the ROC curve was assessed using the bootstrap cross‐validation method. Consequently, the data were resampled with replacement, and for each resample a logistic regression model with the same risk factor as the final model (assuming the final model to be true) was developed. This method was applied to the full, non‐resampled data set to obtain a validating AUC value. This method was repeated 10,000 times (10,000 replicates). The width of the 95% range of the validating AUCs of the resamples (2.5% and 97.5% quantiles for AUCs) was used as the internal validity measure.

## Results

### Patient characteristics and perioperative data

A total of 268 patients underwent major hepatectomy from January 2008 to December 2018, of whom 38 (14.6%) had IAI. The overall mortality rate was 4.85% (13/268). For the analyses, the patients were divided into two groups: IAI (*n* = 38) and non‐IAI (*n* = 230). The clinicopathological characteristics of the two groups are shown in Table 1. The IAI group contained more patients with a higher ASA class (class IV) (16.22% vs. 4.82%, *P* = 0.034), more patients who had undergone previous biliary intervention (34.11% vs. 12.17%, *P* < 0.001), a greater aspartate aminotransferase level (57 vs. 36.5 U/l, *P* = 0.003), a longer operative duration (512 vs. 395 min, *P* < 0.001), greater blood loss (1,800 vs. 1,000 ml, *P* < 0.001), and a greater length of hospital stay (LOH) (24 vs. 11 days, *P* < 0.001) than the non‐IAI group. The IAI group also had a lower hemoglobin level (12.1 vs. 12.9 g/dl, *P* = 0.015) and serum albumin level (33.1 vs. 37.7 g/l, *P* < 0.001) than the non‐IAI group. The IAI group had higher rates of reoperation (13.16% vs. 0.44%, *P *< 0.001), readmission (15.79% vs. 0.87%, *P* < 0.001), and mortality (15.79% vs. 3.04%, *P* = 0.001).

**Table 1:** Patients’ perioperative characteristics

Data	Total (*n* = 268)	Non‐IAI (*n* = 230)	IAI (*n* = 38)	*P*‐value
Sex				
Male	156 (58.21)	135 (58.70)	21 (55.26)	0.691
Female	112 (41.79)	95 (41.30)	17 (44.74)	
Age, years	54.82 ± 12.61	54.42 ± 12.96	57.28 ± 10.08	0.194
BMI (kg/m^2^), mean ± SD	23.82 ± 3.69	23.84 ± 3.64	23.67 ± 4.08	0.798
Comorbidities				
DM	59 (22.01)	49 (21.30)	10 (26.32)	0.490
Hypertension	97 (36.19)	82 (35.65)	15 (39.47)	0.650
Dyslipidemia	33 (12.31)	28 (12.17)	5 (13.16)	0.864
CKD	2 (0.75)	2 (0.87)	0	0.999
Viral hepatitis	63 (23.51)	55 (23.91)	8 (21.05)	0.700
COPD	6 (2.24)	6 (2.61)	0	0.314
Cirrhosis	10 (3.73)	8 (3.48)	2 (5.26)	0.591
ASA class, *n* = 265				
I	25 (9.43)	24 (10.53)	1 (2.70)	0.034
II	98 (36.98)	84 (36.84)	14 (37.84)	
III	125 (47.17)	109 (47.81)	16 (43.24)	
IV	17 (6.42)	11 (4.82)	6 (16.22)	
Smoking	99 (36.94)	85 (36.96)	14 (36.84)	0.989
Cholangitis	6 (2.24)	6 (2.61)	0	0.599
Previous biliary intervention	41 (15.30)	28 (12.17)	13 (34.11)	0.000
Platelet count ×10^3^, *n* = 265	248 (87, 703)	244 (87, 669)	284 (108, 703)	0.168
Hb, g/dl, *n* = 261	12.8 (5.7, 40.9)	12.9 (5.7, 40.9)	12.1 (7.9, 15.1)	0.015
BUN, mg/dl, *n* = 256	12 (4, 43)	12 (4, 43)	13 (8, 40)	0.245
AST, U/l, *n* = 254	37 (8, 378)	36.5 (8, 378)	57 (10, 220)	0.003
ALT, U/l, *n* = 253	43 (10, 229)	41 (10, 229)	58.5 (13, 221)	0.000
Cholesterol, mg/dl, *n* = 210	199.30 ± 48.85	203.02 ± 49.67	174.46 ± 44.34	0.004
eGFR, *n* = 258	91.53 (4.55, 133.50)	92.05 (4.55, 133.5)	80.00 (30.34, 129.96)	0.077
PT, *n* = 239	12.10 (9.70, 27.00)	12.00 (9.70, 27.00)	12.90 (10.00, 18.10)	0.015
TB, mg/dl, *n* = 242	0.6 (0.2, 24.7)	0.6 (0.2, 24.7)	0.7 (0.2, 5.1)	0.293
PNI, *n* = 214	93.78 ± 34.36	94.34 ± 34.85	90.50 ± 31.65	0.566
PLR, *n* = 217	140..35 (49.49, 625.07)	137.75 (49.49, 587.32)	155.26 (63.98, 625.07)	0.222
NLR, *n* = 217	2.14 (0.57, 30.66)	2.14 (0.57, 30.67)	2.46 (0.80, 21.5)	0.376
Albumin, g/l, *n* = 261	37.2 (21.7, 49)	37.7 (22.2, 49)	33.1 (21.7, 45.7)	0.000
≥35	173 (66.28)	156 (69.64)	17 (45.95)	0.005
<35	88 (33.72)	68 (30.36)	20 (54.05)	
ICG‐R15, *n* = 110	11.40 (0.1, 53.8)	11.3 (0.1, 53.8)	11.9 (1.9, 36.6)	0.416
NAF score, *n* = 267				
A (normal‐mild malnutrition)	192 (71.91)	167 (72.93)	25 (65.79)	0.365
B (moderate malnutrition)	75 (28.09)	62 (27.07)	13 (34.21)	
Diagnosis				
Hepatocellular carcinoma	84 (31.34)	73 (31.74)	11 (28.95)	0.144
Cholangiocarcinoma	89 (33.21)	69 (30.00)	20 (52.63)	
Liver metastasis	50 (18.66)	45 (19.57)	5 (13.16)	
Hepatolithiasis	3 (1.12)	2 (0.87)	1 (2.63)	
Liver abscess	1 (0.37)	1 (0.43)	0	
Liver donor	22 (8.21)	22 (9.57)	0	
Benign hepatic tumor	10 (3.73)	9 (3.91)	1 (2.63)	
Gallbladder cancer	1 (0.37)	1 (0.43)	0	
Hepatic cyst	8 (2.99)	8 (3.48)	0	
Biliary reconstruction, *n* = 265	4 (1.51)	3 (1.32)	1 (2.63)	0.464
Operative time, min	411.30 ± 150.69	395.16 ± 145.78	512.63 ± 143.09	0.000
Blood loss, ml	1,200 (50, 32,000)	1,000 (50, 3,200)	1,800 (300, 16,000)	0.000
Antibiotic coated suture material, *n* = 220				
Antibiotic coated	206 (93.64)	178 (93.19)	28 (96.55)	0.700
Non‐antibiotic coated	14 (6.36)	13 (6.81)	1 (3.45)	
Closure suture material, *n* = 219				
Monofilament	96 (43.84)	79 (41.36)	17 (60.71)	0.054
Multifilament	123 (56.16)	112 (58.64)	11 (39.29)	
Bile leakage				
No	235 (87.69)	205 (89.13)	30 (78.95)	0.077
Yes	33 (12.31)	25 (10.87)	8 (21.05)	
Treatment, *n* = 267				
Stitch off	7 (2.62)	5 (2.18)	2 (5.26)	0.271
PCD	18 (6.74)	5 (2.18)	13 (34.21)	0.000
Reoperation	6 (2.25)	1 (0.44)	5 (13.16)	0.000
LOH, days	11 (3, 106)	11 (3, 106)	24.5 (7, 90)	0.000
Readmission	8 (2.99)	2 (0.87)	6 (15.79)	0.000
Death	13 (4.85)	7 (3.04)	6 (15.79)	0.001

Data are presented as *n* (%), mean ± standard deviation, or median (range)

*ALT* alanine aminotransferase, *ASA* American Society of Anesthesiologists, *AST* aspartate aminotransferase, *BMI* body mass index, *BUN* blood urea nitrogen, *COPD* chronic obstructive pulmonary disease, *DM* diabetes mellitus, *eGFR* estimated glomerular filtration rate, *Hb* hemoglobin, *IAI* intra‐abdominal infection, *ICG‐R15* indocyanine green retention test at 15 min, *LOH* length of hospital stay, *NAF* nutritional alert form, *NLR* neutrophil‐to‐lymphocyte ratio, *PCD* percutaneous drainage, *PLR* platelet‐to‐lymphocyte ratio, *PNI* prognostic nutritional index, *PT* prothrombin time, *TB* total bilirubin

Regarding to the preoperative serum albumin, the preoperative characteristics of patients associated with normal and low serum albumin level are displayed in Table 2. The low serum albumin group contained more patients with higher ASA class (class IV) (12.6 vs. 3.47%, *P* < 0.001), higher smoking populations (47.7 vs. 32.9%, *P* = 0.020), more patients who had undergone previous biliary intervention (29.5 vs. 8.09%, *P* < 0.001), higher platelet level (294 vs. 234 × 10^3^, *P* < 0.001), higher aspartate aminotransferase level (42.5 vs. 34.5, *P* = 0.001), longer prothrombin time (12.8 vs. 11.8, *P* < 0.001), higher serum total bilirubin level (1.07 vs. 1.02 mg/dl, *P* = 0.008), greater malignant patients (86.3 vs. 72.2%, *P* = 0.010) than normal serum albumin group. The low serum albumin group also had lower hemoglobin level (11.8 vs. 13.1 g/dl, *P* < 0.001).

**Table 2:** Patients’ perioperative characteristics compared between normal and low serum albumin groups

Data	Total (*n* = 261)	Albumin ≥35 (*n* = 173)	Albumin <35 (*n* = 88)	*P*‐value
Sex				
Male	153 (58.62)	95 (54.91)	58 (65.91)	0.088
Female	108 (41.38)	78 (45.09)	30 (34.09)	
Age, years	55.01 ± 12.42	54.26 ± 13.18	56.46 ± 10.70	0.148
BMI	23.8 ± 3.68	23.92 ± 3.47	23.76 ± 4.11	0.746
Comorbidities				
DM	59 (22.61)	36 (20.81)	23 (26.14)	0.331
Hypertension	96 (36.78)	62 (35.84)	34 (38.64)	0.658
Dyslipidemia	32 (12.26)	21 (12.14)	11 (12.50)	0.933
Chronic kidney disease	2 (0.77)	2 (1.16)	0	0.551
Viral hepatitis	61 (23.37)	41 (23.70)	20 (22.73)	0.861
COPD	5 (1.92)	3 (1.73)	2 (2.27)	0.764
Cirrhosis	10 (3.83)	7 (4.05)	3 (3.41)	0.800
ASA class, *n* = 260				
I	24 (9.23)	23 (13.29)	1 (1.15)	0.000
II	95 (36.54)	72 (41.62)	23 (26.44)	
III	124 (48.69)	72 (41.62)	52 (59.77)	
IV	17 (6.54)	6 (3.47)	11 (12.64)	
Smoking	99 (37.93)	57 (32.95)	42 (47.73)	0.020
Cholangitis	6 (2.30)	3 (1.73)	3 (3.41)	0.408
Previous biliary intervention	40 (15.33)	14 (8.09)	26 (29.55)	0.000
Platelet count ×10^3^, *n* = 260	247 (87, 703)	234 (102, 630)	294 (87, 703)	0.000
Hb, g/dl, *n* = 257	12.8 (5.7, 40.9)	13.1 (5.7, 40.9)	11.8 (7.9, 40.9)	0.000
BUN, mg/dl, *n* = 252	12 (4, 43)	13 (4, 43)	12 (5, 34.8)	0.147
AST, U/l, *n* = 260	37 (8, 378)	34.5 (8, 378)	42.5 (10, 235)	0.001
ALT, U/l, *n* = 259	43 (10, 229)	41 (12, 229)	50 (10, 224)	0.105
Cholesterol, mg/dl, *n* = 215	199.19 ± 49.97	202.90 ± 50.52	191.91 ± 48.39	0.132
eGFR, *n* = 258	91.04 (4.55, 133.5)	91.61 (4.55, 133.50)	89.49 (33.60, 129.96)	0.992
PT, *n* = 239	12.10 (9.70, 27.00)	11.8 (9.70, 27.00)	12.80 (10.80, 26.1)	0.000
TB, mg/dl, *n* = 241	0.6 (0.2, 21.4)	0.6 (0.2, 12)	0.7 (0.2, 21.4)	0.008
PNI, *n* = 214	93.78 ± 34.36	96.27 ± 33.84	88.56 ± 35.11	0.125
PLR, *n* = 214	140.23 (49.49, 625.07)	128.06 (54.55, 587.32)	176.69 (49.49, 625.07)	0.000
NLR, *n* = 214	2.15 (0.57, 30.67)	1.96 (0.57, 30.67)	2.62 (0.84, 21.50)	0.001
IAI				
No	224 (85.82)	156 (90.17)	68 (77.27)	0.005
Yes	37 (14.18)	17 (9.83)	20 (22.73)	
ICG‐R15, *n* = 110	11.4 (0.1, 53.8)	11.3 (0.1, 29.5)	12.2 (0.1, 53.8)	0.811
NAF score, *n* = 261				
A (normal‐mild malnutrition)	186 (71.26)	129 (74.57)	57 (64.77)	0.098
B (moderate malnutrition)	75 (28.74)	44 (25.43)	31 (35.23)	
Diagnosis				
Hepatocellular carcinoma	82 (31.42)	51 (29.48)	31 (35.23)	0.015
Cholangiocarcinoma	87 (33.33)	50 (28.90)	37 (42.05)	
Liver metastasis	50 (19.16)	35 (20.23)	15 (17.05)	
Hepatolithiasis	3 (1.15)	2 (1.16)	1 (1.14)	
Liver abscess	1 (0.38)	0	1 (1.14)	
Liver donor	20 (7.66)	19 (10.98)	1 (1.14)	
Benign hepatic tumor	10 (3.83)	9 (5.20)	1 (1.14)	
Gallbladder cancer	–	–	–	–
Hepatic cyst	8 (3.07)	7 (4.05)	1 (1.14)	
Biliary reconstruction, *n* = 260	4 (1.54)	4 (2.33)	0	0.303
Operative time, min	411.78 ± 152.03	382.94 ± 136.75	469.13 ± 1164.94	0.000
Blood loss, ml	1,200 (50, 32,000)	1,000 (50, 16,000)	1,600 (100, 32,000)	0.000
Antibiotic coated suture material, *n* = 217				
Antibiotic coated	203 (93.55)	137 (94.48)	66 (91.67)	0.427
Non‐antibiotic coated	14 (6.45)	8 (5.52)	6 (8.33)	
Closure suture material, *n* = 216				
Monofilament	94 (43.52)	60 (41.38)	34 (47.89)	0.365
Multifilament	122 (56.48)	85 (58.62)	37 (52.11)	
Bile leakage				
No	228 (87.36)	150 (86.71)	78 (88.64)	0.657
Yes	33 (12.64)	23 (13.29)	10 (11.36)	
Treatment				
Stitch off, *n* = 261	7 (2.68)	6 (3.47)	1 (1.14)	0.270
PCD, *n* = 261	17 (6.51)	11 (6.36)	6 (6.82)	0.887
Reoperation, *n* = 267	6 (2.30)	2 (1.16)	4 (4.55)	0.194
LOH, days	11 (1, 106)	10 (1, 106)	15 (1, 90)	0.000
Readmission	8 (3.07)	6 (3.47)	2 (2.27)	0.721
Death	12 (4.60)	2 (1.16)	10 (11.36)	0.000

Data are presented as *n* (%), mean ± standard deviation, or median (range)

*ALT* alanine aminotransferase, *ASA* American Society of Anesthesiologists, *AST* aspartate aminotransferase, *BMI* body mass index, *BUN* blood urea nitrogen, *COPD* chronic obstructive pulmonary disease, *DM* diabetes mellitus, *eGFR* estimated glomerular filtration rate, *Hb* hemoglobin, *IAI* intra‐abdominal infection, *ICG‐R15* indocyanine green retention test at 15 min, *LOH* length of hospital stay, *NAF* nutritional alert form, *NLR* neutrophil to lymphocyte ratio, *PCD* percutaneous drainage, *PLR* platelet‐to‐lymphocyte ratio, *PNI* prognostic nutritional index, *PT* prothrombin time, *TB* total bilirubin

### Analysis of risk factors associated with IAI

The results of the univariate and multivariate analyses of potential predictors of IAI are shown in Table 3. The univariate analysis identified the following variables as significantly associated with IAI: ASA class IV (OR 13.0; 95% CI 1.40–122.2; *P* < 0.05), previous biliary intervention (OR 3.75; 95% CI 1.72–8.16; *P* < 0.01), hemoglobin concentration (OR 0.81; 95% CI 0.67–0.98; *P* < 0.05), serum albumin concentration (OR 0.887; 95% CI 0.83–0.94; *P* < 0.01), operative time (OR 1.612; 95% CI 1.28–2.02; *P* < 0.01), and blood loss (OR 1.153; 95% CI 1.03–1.28; *P* < 0.01). In the multivariate analysis, only the operative time (OR 1.506; 95% CI 1.18–1.91; *P* < 0.01) and serum albumin concentration (OR 0.911; 95% CI 0.84–0.97; *P* < 0.05) were significantly associated with IAI.

**Table 3:** Univariate and multivariate analysis of predictors of intra‐abdominal infection

Data	Univariate		Multivariate	
	OR (95% CI)	*P*‐value	OR (95% CI)	*P*‐value
Sex				
Male	1			
Female	1.150 (0.57–2.29)	0.691		
Age, years	1.018 (0.99–1.05)	0.195		
BMI	0.987 (0.89–1.08)	0.798		
Comorbidities				
DM	1.319 (0.59–2.90)	0.491		
Hypertension	1.177 (0.58–2.38)	0.650		
Dyslipidemia	1.093 (0.39–3.03)	0.864		
Chronic kidney disease	–	–		
Viral hepatitis	0.848 (0.36–1.95)	0.700		
COPD	–	–		
Cirrhosis	1.541 (0.31–7.55)	0.593		
ASA class				
I	1			
II	4.000 (0.50–31.98)	0.191		
III	3.522 (0.44–27.86)	0.233		
IV	13.090 (1.40–122.2)	0.024		
Smoking	0.995 (0.48–2.02)	0.989		
Cholangitis	–	–		
Previous biliary intervention	3.751 (1.72–8.16)	0.001		
Platelet count x10^3^	1.259 (0.92–1.70)	0.136		
Hb, g/dl	0.814 (0.67–0.98)	0.035		
BUN, mg/dl	1.038 (0.97–1.10)	0.201		
AST, U/l	1.006 (0.99–1.02)	0.050		
ALT, U/l	1.009 (1.00–1.02)	0.023		
Cholesterol, mg/dl	0.987 (0.97–0.99)	0.006		
eGFR	0.985 (0.97–1.00)	0.068		
PT	1.130 (0.96–1.32)	0.124		
TB, mg/dl	0.980 (0.84–1.14)	0.794		
PNI	0.996 (0.98–1.01)	0.564		
PLR	1.291 (0.88–1.88)	0.185		
NLR	1.066 (0.96–1.18)	0.223		
Albumin, g/l	0.887 (0.83–0.94)	0.000	0.910 (0.83–0.99)	0.031
ICG‐R15	1.338 (0.91–1.95)	0.134		
NAF score				
A	1			
B	1.400 (0.67–2.91)	0.366		
Preoperative diagnosis				
Benign	1			
Malignant	1.828 (0.61–5.46)	0.280		
Biliary reconstruction	2.018 (0.20–19.92)	0.548		
Operative time, min	1.612 (1.28–2.02)	0.000	1.580 (1.19–2.09)	0.001
Blood loss, ml	1.153 (1.03–1.28)	0.008		
Antibiotic coated suture material				
Antibiotic coated	1			
Non‐antibiotic coated	0.489 (0.06–3.88)	0.499		
Closure suture material				
Monofilament	1	0.058		
Multifilament	0.456 (0.20–1.03)			
Bile leakage				
No	1			
Yes	2.186 (0.90–5.29)	0.083		

*ALT* alanine aminotransferase, *ASA* American Society of Anesthesiologists, *AST* aspartate aminotransferase, *BMI* body mass index, *BUN* blood urea nitrogen, *CI* confidence interval, *COPD* chronic obstructive pulmonary disease, *DM* diabetes mellitus, *eGFR* estimated glomerular filtration rate, *Hb* hemoglobin, *IAI* intra‐abdominal infection, *ICG‐R15* indocyanine green retention test at 15 min, *LOH* length of hospital stay, *NAF* nutritional alert form, *NLR* neutrophil to lymphocyte ratio, *OR* odds ratio, *PCD* percutaneous drainage, *PLR* platelet‐to‐lymphocyte ratio, *PNI* prognostic nutritional index, *PT* prothrombin time, *TB* total bilirubin

### Predictors of IAI based on preoperative serum albumin level and operative duration

**Figure 1:**
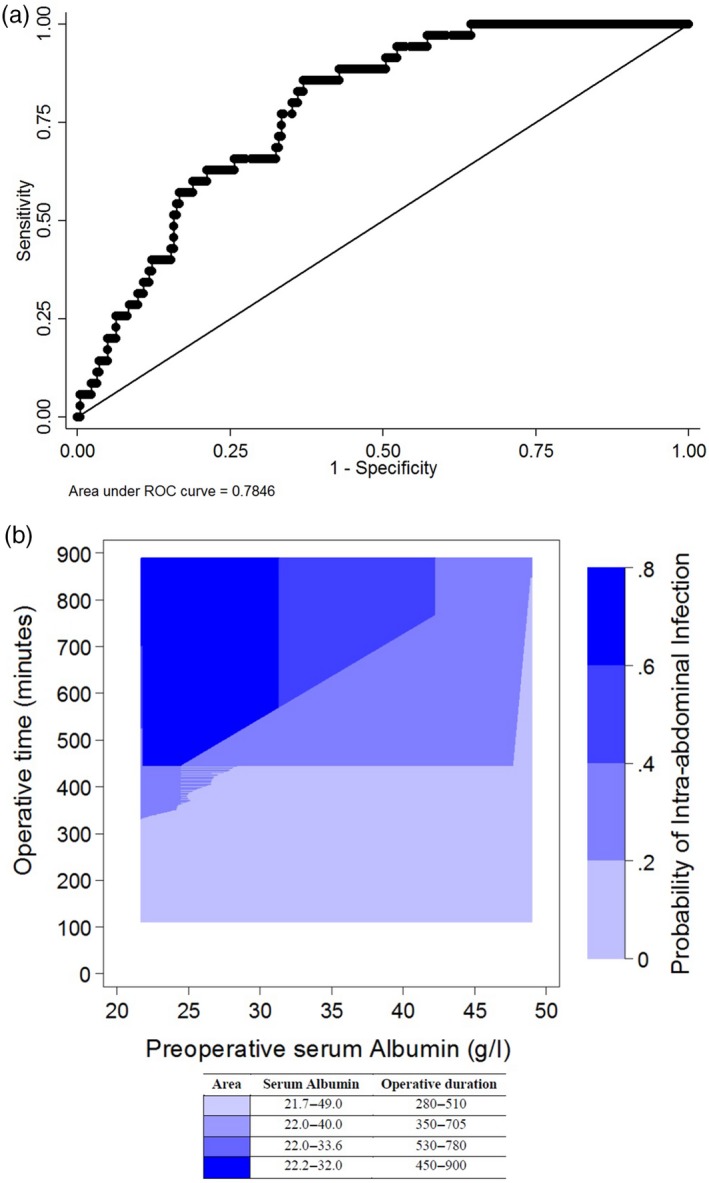
(**a**) Receiver operating characteristic curve for predicting intra‐abdominal infection based on the serum albumin level and operative duration. The area under the receiver operating characteristic curve is 0.7846. (**b**) This figure shows a contour plot the estimated probability of intra‐abdominal infection following major hepatectomy stratified according to the serum albumin concentration and operative duration, based on the logistic regression model. As an example, the darkest blue shaded are in the upper left had corner of the figure represents probabilities of intra‐abdominal infection between 0.6–0.8, in which the operative duration ranges between 450–900 min, and the preoperative albumin level ranges between 22–32 g/l

The performance of the final prediction model of important risk factors for IAI was examined in 268 patients. When the final model was fitted using total logistic regression, the operative time and albumin concentration remained in the final model. According to our final prediction model, the predictive performance of the serum albumin level and operative duration for the risk of IAI based on 268 patients was shown by an AUC of 0.7846 (Fig. 1a). Moreover, a logistic regression model to estimate the probability of IAI based on preoperative serum albumin and operative duration in the 298 patients was constructed from those risk factors as described in the following formula:

$$\text{Predicted}\;\text{probability}=0.683+1.506\left(\text{operative}\;\text{time}\right)+0.911\left(\text{albumin}\right).$$

The performance of the final model for the probability of IAI in 268 patients in terms of the AUC showed the AUC to be 0.785, standard error, 0.035 (Fig. 1). Calculations for the Bootstrap cross‐validation showed the 2.5–97.5% quantiles (95%) interval of the 10,000 resamples to be between 0.741 and 0.787, which included the AUC of the full data (0.785). The width of this interval is only 0.046 and can be considered sufficiently narrow (of the same order as the standard error of the AUC estimate), confirming the validity of the final model.

## Discussion

Major hepatectomy, which is defined as the removal of four or more liver segments, is a complex surgery associated with a high morbidity rate when compared with minor hepatectomy 5, 6, 7, 14. Previous studies have demonstrated that postoperative morbidity is associated with an increased LOH, a higher reoperation rate, a subsequently higher cost of the inpatient stay, and poor long‐term outcomes 23, 24. Our study results are consistent with these previous reports in that the LOH, reoperation rate, and mortality rate were significantly higher in the IAI group. Infectious complications following hepatectomy are common; according to one report, the incidence rate may reach 20% 13. A common infectious complication is IAI or organ/space SSI, and the long‐term outcomes are affected by this complication 13, 14. The mechanisms underlying the poor long‐term outcomes of patients with postoperative IAI are postulated to involve the inflammatory process. Postoperative infections are associated with excessive and sustained levels of proinflammatory cytokine release, and cell‐mediated immunity is suppressed by the effects of anesthesia and the postoperative stress response, which in turn increases the growth of occult micrometastasis 25. In the present study, the incidence of IAI following major hepatectomy was 14%, which is comparable with previous studies 10, 15. However, the incidence of IAI in our study was relatively higher than previous studies from Moreno Elola‐Olaso et al. 11 and Kenjo et al. 2. This may be explained by differences in the patient characteristics and operative time. The preoperative serum albumin in our study was lower (3.7 vs. 4.0 g/dl) and longer operative duration (411 vs. 253 min) than previous two studies. Moreover, patients in our study had a higher proportion of low serum albumin group (33.7 vs. 16%) than previous studies.

From our study, the multivariate analysis showed that the independent factors associated with IAI following major hepatectomy were the preoperative serum albumin level and the operative time. Serum albumin is a negatively charged protein that is synthesized by hepatocytes and released into the circulation, and the rate of albumin synthesis by hepatocytes is regulated by changes in the plasma colloid pressure, the metabolic and inflammatory state of the host, and various anabolic and catabolic hormones 26, 27. Serum albumin has been used as a surrogate marker of patients’ nutritional state worldwide. Fuhrman et al. 27 reported that inflammation exerts the most significant effects on the serum protein level by altering normal hepatic protein metabolism and inducing capillary leakage. They concluded that hepatic proteins are indicators of morbidity and mortality and recovery from acute and chronic disease. The preoperative serum albumin is strongly associated with postoperative complications in patients undergoing hepatectomy 4. However, the association of preoperative hypoalbuminemia as a predictor of postoperative IAI following hepatectomy is controversial. Moreover, most studies have included both minor and major hepatectomy 13, 23. In the present study, all patients underwent major hepatectomy, which is a complex surgery with a high morbidity rate. Our results showed that the serum albumin level was a significant independent factor associated with IAI. Regarding to the preoperative hypoalbuminemia (<35 g/l) patients had significantly greater incidence of IAI following major hepatic resection than normal groups. This result is consistent with previous reports from two large cohort studies from the United States. Moreno Elola‐Olaso et al. 11 performed a large‐population retrospective study of the predictors of SSI after liver resection using the American College of Surgeons National Surgical Quality Improvement Program (ACS‐NSQIP) database, and the results showed that serum albumin was a predictor of SSI (both superficial and deep or organ/space SSI). Neumayer et al. 28 performed a large‐population study and found that among 163,624 patients undergoing vascular and general surgical procedures, low serum albumin levels of <3.5 mg/dl was independent risk factors associated with increased risk of SSI. According to these reports, the preoperative serum albumin level is strongly predictive of postoperative IAI following hepatectomy, especially major hepatectomy. Hypoalbuminemia, which is commonly observed in malnutrition patients is associated with a series of physiological derangement that may lead to complications and death 29. Consequently, the malnourished patients who are undergoing major operations are at significant risk from perioperative complications such as infectious complications 30. The hypoalbuminemia associated with IAI following hepatectomy is explained by alterations in both innate and adaptive immune function contribute significantly to increased susceptibility to infections 31. Moreover, protein wasting during the postoperative period is believed to represent the metabolic cost of rapid mobilizing amino acids for wound healing and the synthesis of immune cells and proteins 32. The recent guideline from European Society for Clinical Nutrition and Metabolism (ESPEN) state that nutrition is critically important to recovery from major surgery and patients at a high risk due to impaired nutritional status should ideally receive oral supplementation prior to major surgery, even if this results in the delay of resection of a malignancy 33. Nevertheless, the serum albumin following preoperative nutritional intervention is not considered as a marker for well‐nourished patients, because of the nutritional support often fails to improve serum albumin levels 29. To the best of our knowledge, no randomized, controlled trial study has yet been performed to evaluate the increase of the preoperative serum albumin level with IAI in patients undergoing major hepatectomy. Thus, a further well‐designed prospective randomized controlled trial study comparing low and high serum albumin patients undergoing major hepatectomy should be conducted.

Our study also showed that the operative time was an independent factor associated with IAI following major hepatectomy. A prolonged operative time is associated with an increased risk of postoperative SSI 34, 35. Procter et al. 34 performed a retrospective study of 299,359 operations performed at 173 hospitals from the ACS‐NSQIP and found that the infectious complication rate increased linearly with the operative duration. They concluded that the operative duration is independently associated with increased infectious complications and the LOH after adjustment for procedure‐ and patient‐related risk factors 34. A prolonged operative time is associated with increased exposure of microbes to the environment, heightened surgical stress to the immune system, and diminished efficacy of antimicrobial prophylaxis over time 13, 35. Several studies have shown that the operative time is a significant independent factor associated with SSI, including organ/space SSI, in patients undergoing hepatectomy 11, 12. Determinants of the operative duration are multifactorial and include the operative type, proficiency of the surgeon, presence of surgical trainees, communication among operative professionals, and whether the operation is performed on an emergency basis 36. Major hepatectomy usually has a long operative time because of the complexity of the operation. With respect to improvements in the efficacy of the procedure to reduce the operative duration, Procter et al. 34 and Haynes et al. 37 recommended improvements in team communication and the consistency of operating room staffing using a surgical safety checklist program, technology, and process efficiency to reduce the operative duration.

This study had some limitations. First, because of its retrospective nature, some selection bias may have been present. Second, the population of our study was relatively small when compared with previous studies.

## Conclusion

Major hepatectomy is associated with a high morbidity rate. The preoperative serum albumin level is an important marker of patients’ nutritional and inflammatory status. The present study indicates that the serum albumin level and operative duration are significant predictors of postoperative IAI. In patients with a low serum albumin level who are undergoing major hepatectomy, the surgeon should consider preoperative nutritional intervention with the use of a short‐term high‐protein supplement diet and closely monitor the patients for the development of postoperative IAI. A further prospective study of perioperative nutritional support to improve treatment outcomes in such patients should be conducted.

## Conflict of interest

None declared.
